# Perceptions and preparedness toward tobacco cessation counseling amongst clinical medical students in Chongqing, Southwest China: A cross-sectional study

**DOI:** 10.3389/fpubh.2022.934782

**Published:** 2022-08-01

**Authors:** Chuang Yang, Wenjin He, Ruihang Deng, Mohan Giri, Haiyun Dai

**Affiliations:** ^1^The First College of Clinical Medicine, Chongqing Medical University, Chongqing, China; ^2^Department of Respiratory and Critical Care Medicine, The First Affiliated Hospital of Chongqing Medical University, Chongqing, China

**Keywords:** tobacco education, clinical experience, smoking cessation counseling, medical students, medical education

## Abstract

**Background:**

Medical students play an indispensable role in providing smoking cessation counseling. Despite the rapid increase in tobacco use, there is little data on what Chinese medical students know or are taught about it. This study aims to investigate the relationship between medical students' tobacco education level, clinical experience, and tobacco cessation counseling (TCC) provided by medical students.

**Methods:**

This cross-sectional study was carried out among clinical medical students of Chongqing medical university. An anonymous, self-administered questionnaire included items on demographic information, perceptions, and perceived preparedness, clinical medical students' self-reported level of education about alternative tobacco products, and traditional cigarettes. We assessed their perspectives toward TCC using a 5-point Likert scale. Descriptive and binary logistic regression analyses were carried out.

**Results:**

A total of 1,263 medical students completed the questionnaire. The majority of students (85%) expressed a willingness to provide TCC to patients in need. However, only half of the students stated unequivocally that they knew some ways and methods of tobacco cessation, while 18% stated that they did not know methods of tobacco cessation. Tobacco education and clinical experience were significantly associated with the ability to provide TCC. Our findings revealed that students with more clinical experience (undergraduates: B = 0.326, *P* < 0.001; postgraduates: B = 0.518, *P* < 0.001) were significantly more likely to have a greater self-reported comprehensive ability to provide TCC.

**Conclusion:**

Tobacco education and clinical experience can enhance the ability of medical students to provide smoking cessation counseling. There is a need to focus on alternative tobacco products with changing times, and curriculum planners should collaborate to incorporate comprehensive tobacco prevention and cessation training into the medical school curriculum.

## Introduction

According to the world health organization (WHO), tobacco use is the leading cause of cancer and preventable death, accounting for over 8 million deaths per year (1). As the world's largest tobacco consumer, the tobacco epidemic in China is one of the most serious public health threats. China has over 300 million smokers, with male smoking rates as high as 52.1% (2). Moreover, the annual number of deaths due to smoking-related diseases exceeds 1 million, and the number of deaths caused by second-hand smoke exposure exceeds 100,000 (2). However, according to a recent study, the smoking rate is on a downward trend from 2010 to 2018, reflecting an increase in public awareness of the risks of tobacco products and a corresponding increase in public demand for smoking cessation (3). The significant increase in smoking prevalence among young people aged 16–20, from 11.72% in 2010 to 14.29% in 2018, is a public health concern in China (3). Up to now, the hospital smoking cessation clinic and smoking cessation intervention work can provide patients with tobacco cessation counseling (TCC) and treatment effective in treating tobacco use and nicotine addiction. However, there are some potential issues. The current smoking cessation interventions have formed a series of smoking cessation protocols and methods, primarily for patients using traditional cigarettes, whereas there are many gaps in the case of non-traditional or alternative tobacco products (ATPs), such as e-cigarettes and water pipe smoking (4, 5).

Physicians or doctors play an important role in providing patients with tobacco cessation counseling and interventions as smokers regard their physicians as the most trusted source of information (6). Compared with anesthesiology, laboratory medicine students, and so on, clinical medical students in future positions have a greater chance and more time to manage and contact smoking patients (7). The basic education period for clinical medical undergraduates is the best time to receive tobacco cessation counseling training and tobacco medicine education before beginning their careers as physicians. There is a lack of assessment of the self-reported comprehensive ability of clinical medical students providing TCC at Chinese medical universities (8, 9). There is also a lack of training in this area and recommendations and programs to help improve it (10). Data from the China global adult tobacco survey 2018 show that e-cigarette is popular mainly among young people whose access to e-cigarette is through the Internet, and their use, recognition, and publicity are all higher in 2018 than in 2015 (11). Analyzing data from 2018, it is not difficult to predict that the trend of e-cigarette use, recognition, and publicity will increase year after year until 2020 (11). The ensuing health risks of e-cigarettes and the phenomenon of e-cigarette dependence have become hot topics of concern that should not be underestimated. Smoking cessation counseling interventions for alternative tobacco products such as e-cigarettes and water-pipe smoking should be improved and implemented to meet the future needs of alternative tobacco product users to quit smoking (12). In the field of tobacco medicine education, the curriculum system and curriculum development in Chinese medical schools are not ideal. In the compulsory medicine courses, the scattered knowledge of tobacco disease is not conducive to forming overall cognition among medical students. Previous studies (8, 13) also showed that the feedback of medical students who take this course is less-than-desirable, with the main issue being an inability to apply the theoretical knowledge that students learned in class to practice. With the average smoking age in China declining (14), the rising trend of ATPs, and the growing demand from patients to quit smoking, it is practical and necessary to investigate the self-reported comprehensive ability of clinical medical students to provide TCC at different years of study and analyze their differences, sources of differences and influencing factors.

### Research questions

(i) What are the clinical medical students' perceptions and perceived preparedness toward tobacco cessation counseling?
(ii) Are there any differences in clinical medical students' perceptions and perceived preparedness toward tobacco cessation counseling?
(iii) What is the correlation between the self-reported comprehensive ability of clinical medical students providing TCC and self-reported level of education about ATPs and traditional cigarettes or self-reported degree of clinical experience?

## Methods

### Setting and participants

In this cross-sectional study, all the undergraduate clinical medical students and master's students in the first affiliated hospital of Chongqing Medical University, including some students from the second affiliated hospital (estimated number = 1,522), were invited to participate in an anonymous Internet survey. Students majoring in clinical medicine in mainland China need to finish 5 years of medical school studies to obtain a bachelor's degree; the 5th year is the clinical session. The questionnaire remained open for about 2 months, from April to June 2021. An online questionnaire platform was adopted in the study to send a questionnaire and gather the information. For undergraduates, we introduced our project in class and encouraged students to scan the QR code of our questionnaire to complete it during offline class. We distributed QR codes of the survey questionnaire to master students through WeChat groups (WeChat is a Chinese instant messaging, social media, and mobile app). According to the affiliated hospitals, master students have their own WeChat group. Survey QR code was shared in the Master students' WeChat group of the first and second affiliated hospitals of Chongqing Medical University. One can only fill out a survey once with one WeChat account, avoiding duplicate entries. The survey can be submitted only after participants have completed every item in the questionnaire in order to close the webpage, ensuring a 100% completion rate. After finishing the survey, participants can participate in a lucky draw to win red envelopes.

### Survey questionnaire

The questionnaire was developed partly based on several early validated researches (15, 16), but it was modified and contained additional questions to conform to the aim of the study. The survey questionnaire consisted of five major sections: (I) demographic data, (II) perceptions, (III) perceived preparedness, (IV) clinical medical students' self-reported level of education about ATPs and traditional cigarettes, and (V) clinical medical students' self-reported degree of clinical experience. Participants must fill out every item in the questionnaire to close the webpage, ensuring the completion rate is 100%. Responses to survey questions were collected anonymously and de-identified. Students could opt out of the survey at any time.

### Demographic data

All participants were asked to provide information such as their years of study, gender, tobacco use status, and age.

### Perceptions and perceived preparedness

The “perceptions” section of the questionnaire is intended to investigate medical students' views and opinions on tobacco cessation counseling at all stages of study to reflect the degree of potential willingness of medical students to provide tobacco cessation counseling, i.e., the extent to which they are willing to provide tobacco cessation counseling assistance to patients who need to quit smoking. The perceived preparedness section of the questionnaire is designed to investigate the level of knowledge reserved for medical students at all stages of study to provide traditional and non-traditional tobacco cessation counseling as well as the degree of understanding of smoking cessation interventions; to reflect the current stage of medical students; to provide all kinds of smoking cessation counseling, and to manage the degree of self-confidence and readiness of tobacco users. The questionnaire items in these two sections are presented in the form of opinion statements and were answered by undergraduates and master's students using a five-point Likert scale, which ranges from 1 = strongly disagree to 5 = strongly agree.

### Clinical medical students' self-reported level of education about ATPs and traditional cigarettes

Undergraduate students from different years of study were asked to provide the degree of the education they received in tobacco medicine on a five-point scale (1 = no education, 2 = a little education, 3 = some education, 4 = a lot of education, 5 = excessive education). The following questions were designed to assess undergraduates' comprehensive knowledge of alternative and traditional tobacco products: (1) secondhand smoke effects, (2) health effects, and (3) epidemiology.

### Clinical medical students' self-reported degree of clinical experience

For undergraduate students, they were asked to provide information on self-reported experience in observation or volunteering services in local hospitals. Postgraduates of clinical medicine need to answer the question about the degree of personal experience in providing tobacco cessation counseling or managing tobacco-using patients. The survey questions in this section also use a five-point scale, but they differ slightly from the previous ones, as 1 = no experience, 2 = a little experience, 3 = some experience, 4 = a lot of experience, and 5 = excessive experience.

### Data analysis

The online questionnaire was exported to Excel, and the data were imported into IBM SPSS software (version 26.0) for statistical analysis. The Cronbach alpha was used to evaluate the internal consistency of the items constituting the principal component (17). Exploratory factor analysis was used for structural validity analysis to ensure whether factor analysis could be carried out by calculating Kaiser-Meyer-Olkin (KMO) value and Bartlett sphericity test *p*-value (18). Then, the maximum variance method was used for rotation to obtain the rotated component matrix. Descriptive statistical analysis (including the year of study, gender, respondent's tobacco use status, anditems related to cognition and preparation) were carried out using frequencies and percentages. In terms of cognition and preparation at different learning stages, the comparison of secondhand smoke effects, health effects, and epidemiology among different groups was conducted by multi-independent sample non-parametric test (because the data did not follow normal distribution after examination). Analyzing the whole grade differences within the above three topics (secondhand smoke effects, health effects, and epidemiology) by the non-parametric test of two related samples. To verify the hypothesis factors, the bivariate correlation regression analysis model and binary linear regression analysis model were used to calculate the correlation coefficient, regression coefficient, 95% confidence interval (CI), *p*-value, and test level α = 0.05.

### Ethical consideration

This study was conducted according to the ethical guidelines of the Helsinki Declaration (Word Medical Association, 2013). This study was conducted anonymously using an electronic questionnaire *via* social software, and the First Affiliated Hospital of Chongqing Medical University ethics committee ethically approved the study protocol (Approval No: 022-K171). Furthermore, neither human clinical trials nor animal experiments were used in our study. Written informed consent for participation was not required for this study as this study was conducted anonymously.

## Results

### Reliability analysis

The Cronbach alpha of the total scale was 0.852, and the Cronbach alpha of each dimension was between 0.660 and 0.990, indicating that the scale has good reliability and high internal consistency among items.

### Construct validity analysis

Exploratory factor analysis was performed on the scale. The results showed that KMO = 0.860, *X*^2^ = 24142.128 of the Bartlett sphericity test. *P* < 0.001, indicating that factor analysis is suitable. After rotation by maximum variance method. The characteristic root Factors > 1 were extracted as common factors. Three common factors were extracted at the end, corresponding to questions 5–9, 10–14, and question 15–17 in the scale. The cumulative variance contribution rate was 71.206%, that is, the above three factors can explain 71.206% of the content of the questionnaire.

In total, 1,263 students completed the survey, yielding an 82.98% (1,263/1,522) response rate. The response rate for undergraduate students was 91.82% (887/966) and 67.63% (376/556) for master's degree students. Among the 376 master's students who took part in the survey, 70.74% (284/380) were from the first affiliated hospital of Chongqing Medical University, and 52.27% (92/176) were from the second affiliated hospital. Of all the 1,263 respondents from different years of study, 202 (16.0%) were in the 1st year, 201 (16.0%) in the 2nd year, 205 (16.2%) in the 3rd year, 183(14.5%) in the 4th year, 96 (7.6%) in the final year, while 85 (6.7%) were in master degree (MD) program of respiratory and 291 (23%) in MD program of non-respiratory. The mean age was 22.3, ranging from 16 to 38 years; 758 (59.97%) were female, and 506 (40.03%) were male. In terms of tobacco use status, 1,206 (95.4%) participants were non-users, 37 (2.9%) were former users, and 21 (1.7%) were current users (Table 1).

**Table 1:** Students' year of study, their gender and tobacco use status (*n* = 1,263).

**Demographics**	**No. (%)**
**Year of study**	
Year 1	202 (16.0%)
Year 2	201 (15.9%)
Year 3	205 (16.2%)
Year 4	183 (14.5%)
Year 5	96 (7.6%)
MD program of respiratory	85 (6.7%)
MD program of non-respiratory	291(23.0%)
**Gender**	
Female	758 (60.0%)
Male	505 (40.0%)
**Respondent's tobacco use status**	
Non-user	1,205 (95.4%)
Former user	37 (2.9%)
Current user	21 (1.7%)

### Clinical medical students' perceptions and perceived preparedness toward providing tobacco cessation counseling

The distribution of clinical medical students' (including undergraduates and postgraduates) perceptions and perceived preparedness toward providing tobacco cessation counseling for smoking patients on a five-point Likert scale is presented in Table 2, while the mean score of each item with different years of study is shown in Table 3. In terms of the willingness to provide TCC for patients in need as a clinical medical student, the majority of students (85%) expressed relatively positive attitudes-choosing “agree” or “strongly agree.” Moreover, nearly all students (92%) suggested that they would advise patients to quit tobacco use in their future careers. However, only 58% of students were positive about the effect of the TCC provided by a clinical medical student, and 35% were unsure if they could assist patients in quitting smoking. When asked questions about cigarettes and ATPs, 70% of students believed that TCC for patients should include counseling of two aspects: traditional cigarettes and ATPs, indicating a proper awareness among clinical medical students about today's prevalence of ATPs and the corresponding needs for people to quit it. However, for the statement “compared with a traditional tobacco product, alternative tobacco products have the same degree of health hazards,” only 48% of students were in favor of it, and around half of the students (52%) were unsure or disagreed this statement.

**Table 2:** Clinical medical students' perceptions and perceived preparedness toward providing tobacco cessation counseling.

**Item**	**Strongly disagree**	**Disagree**	**Neutral**	**Agree**	**Strongly agree**
**Perceptions (*****n*** **=** **1,263)**					
I am willing to provide TCC to patients as a clinical medical student.	25 (2.0%)	19 (1.5%)	150 (11.9%)	565 (44.7%)	504 (39.9%)
I believe TCC by clinical medical students could assist patients to quit smoking.	17 (1.3%)	71 (5.6%)	446 (35.3%)	487 (38.6%)	242 (19.2%)
I will advise patients to quit tobacco use in my future career.	7 (0.6%)	4 (0.3%)	91 (7.2%)	531 (42.0%)	630 (49.9%)
TCC for patients should include traditional tobacco product (e.g., cigarette) and alternative tobacco product (e.g., e-cigarette, water-pipe smoking).	35 (2.8%)	85 (6.7%)	267 (21.1%)	526 (41.6%)	350 (27.7%)
Compared with traditional tobacco product, alternative tobacco product has the same degree of health hazards.	24 (1.9%)	215 (17.0%)	417 (33.0%)	402 (31.8%)	205 (16.2%)
**Perceived preparedness (*****n*** **=** **1,263)**					
I am knowledgeable enough to explain negative impacts of using cigarette to patients.	29 (2.3%)	171 (13.5%)	496 (39.3%)	472 (37.4%)	95 (7.5%)
I am knowledgeable enough to explain negative impacts of using ATPs (alternative tobacco products) to patients.	60 (4.8%)	271 (21.5%)	607 (48.1%)	262 (20.7%)	63 (5.0%)
I am prepared to help patients who need TCC of traditional cigarette.	23 (1.8%)	172 (13.6%)	487 (38.6%)	485 (38.4%)	96 (7.6%)
I am prepared to help patients who need TCC of ATPs.	41 (3.2%)	246 (19.5%)	561 (44.4%)	338 (26.8%)	77 (6.1%)
I know what the tobacco cessation protocol is.	35 (2.8%)	196 (15.5%)	430 (34.0%)	540 (42.8%)	62 (4.9%)

TCC, tobacco cessation counseling.

**Table 3:** Variations of clinical medical students' Perceptions and perceived preparedness toward providing tobacco cessation counseling (TCC).

**Item**	**Year 1** ^*^	**Year 2** ^*^	**Year 3** ^*^	**Year 4** ^*^	**Year 5** ^*^	**MD of respiratory department** ^*^	**MD of non-respiratory department** ^*^	***p*** **-value**
**Perceptions (*****n*** **=** **1,263)**								
I am willing to provide TCC to patients as a clinical medical student.	4.18	3.94	4.14	4.24	4.23	4.54	4.26	<0.001
I believe TCC by clinical medical students could assist patients to quit smoking.	3.65	3.50	3.56	3.59	3.70	3.98	3.90	<0.001
I will advise patients to quit tobacco use in my future career.	4.31	4.24	4.40	4.46	4.49	4.56	4.47	0.001
TCC for patients should include traditional tobacco product (e.g., cigarette) and alternative tobacco product (e.g., e-cigarette, water-pipe smoking).	3.84	3.85	3.77	3.79	3.91	3.86	3.92	0.350
Compared with traditional tobacco product, alternative tobacco product has the same degree of health hazards.	3.32	3.31	3.29	3.36	3.56	3.55	3.67	<0.001
**Perceived preparedness (*****n*** **=** **1,263)**								
I am knowledgeable enough to explain negative impacts of using cigarette to patients.	2.87	3.18	3.46	3.40	3.42	3.72	3.53	<0.001
I am knowledgeable enough to explain negative impacts of using ATPs (alternative tobacco products) to patients.	2.63	2.95	2.98	2.85	2.99	3.4	3.28	<0.001
I am prepared to help patients who need TCC of traditional cigarette.	3.12	3.26	3.37	3.29	3.39	3.56	3.58	<0.001
I am prepared to help patients who need TCC of ATPs.	2.93	3.07	3.08	2.91	3.16	3.49	3.36	<0.001
I know what the tobacco cessation protocol is.	3.19	3.13	3.37	3.24	3.41	3.62	3.42	<0.001
Sum	34.04	34.43	35.42	35.13	36.26	38.28	37.39	

p-value analyze the differences between different grades in each item by Kruskal-Wallis Test.

* Indicates that data is represented by the average score of different grades.

In the “perceived preparedness” section, we intended to assess the students' confidence and perceived preparedness toward providing TCC. In terms of “explaining the negative impacts of tobacco products to patients,” students showed greater confidence in traditional cigarettes than in ATPs. A similar response was obtained with respect to “the perceived preparedness to help patients who need TCC of traditional cigarettes or ATPs.” When asked about the content of “the tobacco cessation protocols,” only half of the students indicated unequivocally that they knew some ways and methods of tobacco cessation, while around 18% of students stated that they didn't know about this. According to the statistical study of the five-point Likert scale, scores of every question can be added up, and the sum of the scores the respondent received by answering each question, representing the strength or weakness of one's attitude toward the topic questionnaire intends to explore. Therefore, it is logical and feasible to calculate every item's average score for each grade and add the average values of these ten items in grades to get a sum of each grade. The sum of each grade can represent the whole grade's attitude toward tobacco cessation counseling, which to some extent reflects the self-reported comprehensive ability of clinical medical students in different stages of study to provide TCC. There were statically significant differences (*P* < 0.001) in most items of students' perceptions and perceived preparedness toward providing TCC among different years of study (Table 3). By analyzing the sum score of each grade, the self-reported comprehensive ability of students providing TCC shows an upward trend from year 1 to year 5, while in the case of master students, students of respiratory indicate a better self-reported comprehensive ability than students of non-respiratory but both of the groups were better than undergraduates.

### Clinical medical students' self-reported level of education about ATP and traditional cigarettes

In terms of students' self-reported level of education about ATPs and traditional cigarettes, data from all classes showed that undergraduate students received more tobacco medicine education about cigarettes than ATPs in all three aspects (secondhand smoke effects, health effects, and epidemiology) (*P* < 0.0001) (Table 4). Among all aspects, the students received the least education about “epidemiology” in terms of both cigarettes and ATPs. By comparing the mean score of each undergraduate class on each item, we can conclude that students from different years of the study received varying levels of education about cigarettes and ATPs in all aspects. Statistically significant differences were found in most items in Table 4, except for the “health effects” of cigarettes. Regarding secondhand smoke effects from cigarettes, year 3 students reported receiving the most education out of all classes, while year 5 students reported receiving the most education in terms of ATPs. The same pattern was discovered for the health effects of cigarettes and ATPs, with students in years 3 and 5 reporting the most education in cigarettes and ATPs, respectively, among all classes. Regarding the epidemiology of cigarettes and ATPs, year 5 students reported receiving the most education in both aspects among all classes. Generally speaking, undergraduates in the third, fourth, and 5th years tend to acquire a comparatively higher level of education about cigarettes and ATPs than students in the 1st and 2nd years.

**Table 4:** Clinical medical students' self-reported level of education about ATPs and traditional cigarettes.

**Topic**	**Overall**^a^ **(*****n*** = **887)**	***p-*** **value** ^b^	Year 1 students^*^ (***n*** = **202)**	Year 2 students^*^ (***n*** = **203)**	Year 3 Students^*^ (***n*** = **203)**	Year 4 students^*^ (***n*** = **183)**	Year 5 Students^*^ (***n*** = **96)**	***p*****-value** ^c^ **(by class)**
**Second smoke effects**								
Cigarettes	3.46	<0.0001	3.38	3.33	3.61	3.52	3.48	0.0385
ATPs	2.52		2.48	2.48	2.46	2.37	2.83	0.0248
**Health effects**								
Cigarettes	3.68	<0.0001	3.60	3.57	3.78	3.73	3.70	0.1686
ATPs	2.64		2.66	2.51	2.55	2.50	2.96	0.0075
**Epidemiology**								
Cigarettes	3.08	<0.0001	2.66	2.88	3.22	3.32	3.34	<0.0001
ATPs	2.38		2.19	2.34	2.33	2.28	2.77	0.0010

* Means that data are represented by the average score of different grades.

a Means the average for the whole grade p-value.

b Analyze group differences within the different topic and refer to the Wilcoxon Test p-value.

c Analyze group differences on different grades and refer to the Kruskal-Wallis Test.

### Clinical medical students' self-reported degree of clinical experience

**Figure 1:**
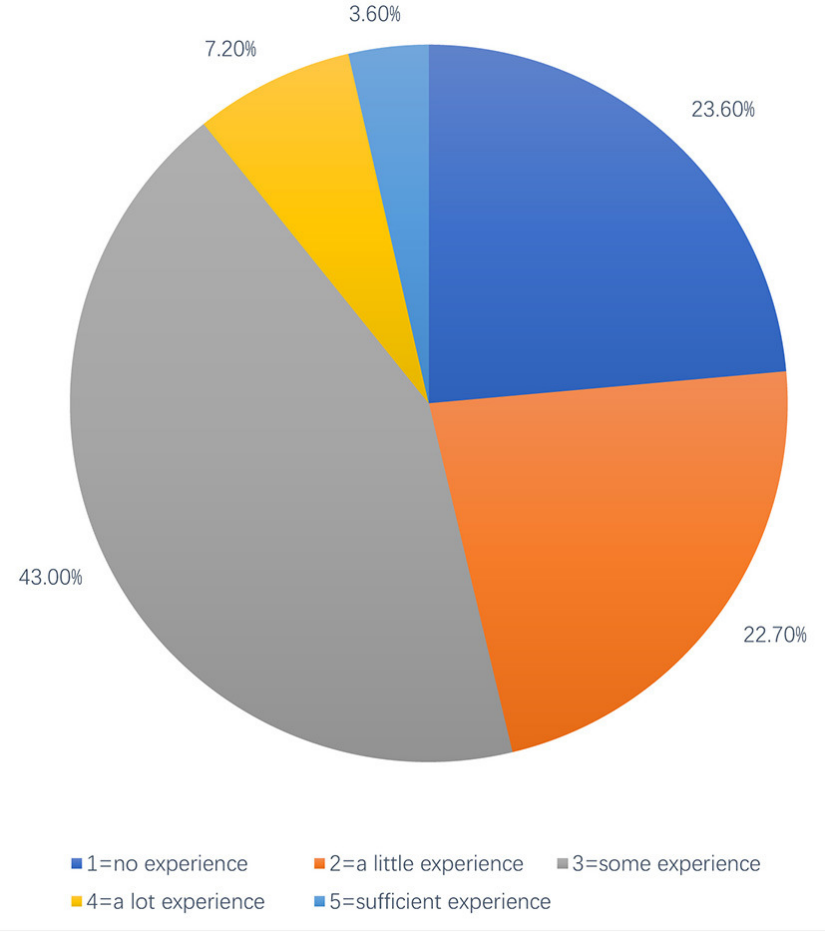
Undergraduate medical students self-reported degree of clinical experience.

**Figure 2:**
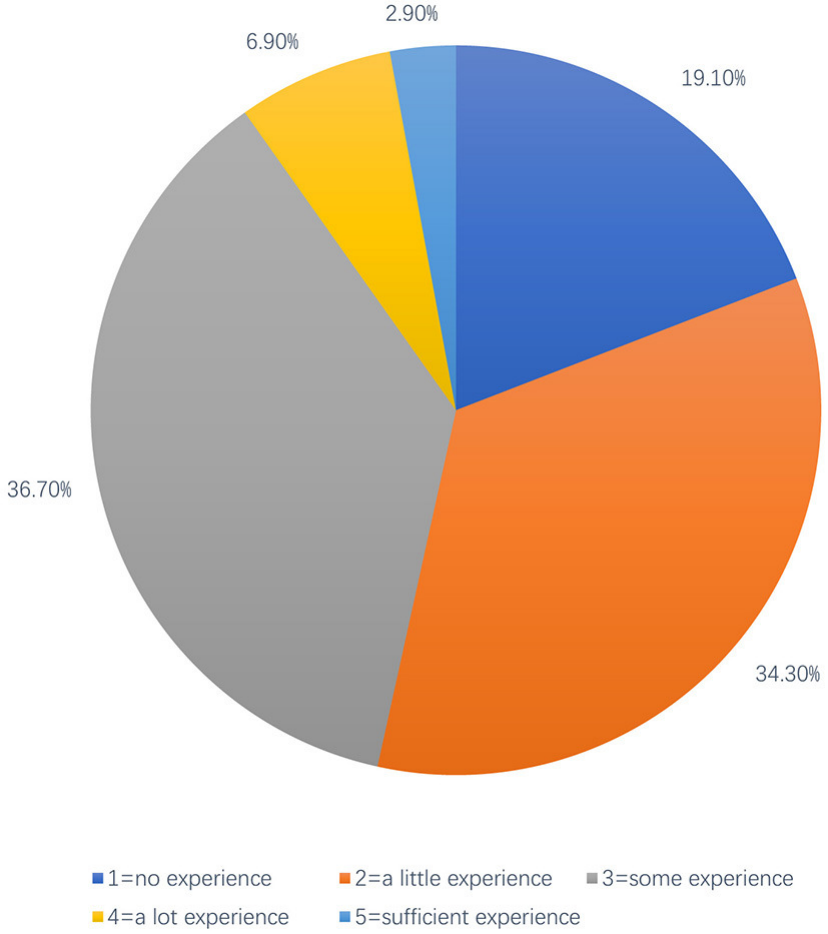
Postgraduate medical students self-reported degree of clinical experience.

In terms of students' self-reported clinical experience level, data from undergraduates (*n* = 887) and postgraduates (*n* = 376) are shown in Figures 1, 2, respectively. Of note, 43.00% of undergraduate respondents reported having some experience of observation or volunteer services in local hospitals, while ~45% reported having little or no clinical experience in hospitals. Only a few of them reported having much clinical expertise (Figure 1). The distribution of the clinical medical postgraduates' self-reported experience in providing TCC or managing tobacco-using patients is similar to the distribution of undergraduates, except for the part of the answer “a little experience,” which is 34.3% for postgraduate respondents (about 12% more than the undergraduate respondents) reported to have little clinical experience related to TCC (Figure 2), probably because the postgraduate respondents included master students of respiratory and non-respiratory, and non-respiratory students may not have the same access to patients with respiratory diseases as a respiratory student.

### Correlation and regression analysis for the self-reported comprehensive ability of clinical medical students providing TCC

We conducted correlation and regression analyses based on the previously collected and processed data. For undergraduates, “the self-reported comprehensive ability of clinical medical students providing TCC” and “self-reported level of education about ATPs and traditional cigarettes” were positively correlated (*r* = 0.427; *p* < 0.001). Furthermore, “the self-reported comprehensive ability of clinical medical students providing TCC” and ““self-reported degree of clinical experience” were also positively correlated *r* = 0.326; *p* < 0.001. For postgraduates, “the self-reported comprehensive ability of clinical medical students providing TCC” and “self-reported degree of clinical experience” were positively correlated (*r* = 0.518; *p* < 0.001). Linear regression analysis results showed that undergraduates with more clinical experience were significantly more likely to possess a greater self-reported comprehensive ability to provide TCC (*R*^2^ = 0.105, *P* < 0.001, B = 0.326, 95% CI: 0.129, 0.191) (Table 5). Furthermore, the results also indicated that undergraduates with a higher self-reported level of education about ATPs and traditional cigarettes were significantly more likely to possess a greater self-reported comprehensive ability to provide TCC (*R*^2^ = 0.182, *P* < 0.001, B = 0.427, 95% CI: 0.201, 0.266). Similarly, the results showed that postgraduates with more clinical experience were significantly more likely to possess a greater self-reported comprehensive ability to provide TCC (*R*^2^ = 0.266, *P* < 0.001, B = 0.518, 95% CI: 0.249, 0.346) (Table 6).

**Table 5:** Correlation and regression analysis for the self-reported comprehensive ability of undergraduate clinical medical students providing TCC.

**A** ***u***	**Regression** **coefficient**	**B**	**Std. error**	***P***	**95% Confidence Interval** **for B (Lower Bound)**	**95% Confidence Interval** **for B (Upper Bound)**	**Beta**
E*u*	2.670	0.233	0.017	<0.001	0.201	0.266	0.427
Adjusted *R* Square					0.182		
*p*-value					<0.001		
*N*					887		
C*u*	3.099	0.160	0.016	<0.001	0.129	0.191	0.326
Adjusted *R* Square					0.105		
*p*-value					<0.001		
N					887		

Au, the self-reported comprehensive ability of clinical medical students providing TCC; Eu, self-reported level of education about ATPs and traditional cigarettes; Cu, self-reported degree of clinical experience; TCC, tobacco cessation counseling.

**Table 6:** Correlation and regression analysis for the self-reported comprehensive ability of graduate clinical medical students providing TCC.

**A** ***p***	**Regression** **coefficient**	**B**	**Std. error**	***P***	**95% Confidence interval** **for B (Lower Bound)**	**95% Confidence interval** **for B (Upper Bound)**	**Beta**
C*p*	3.049	0.296	0.025	<0.001	0.249	0.346	0.518
Adjusted *R* Square					0.266		
*p*-value					<0.001		
N					376		

Ap, the self-reported comprehensive ability of clinical medical students providing TCC; Cp, self-reported degree of clinical experience; TCC, tobacco cessation counseling.

## Discussion

To the best of our knowledge, our study is the first to assess the perceived preparedness and self-reported comprehensive ability of Chinese medical students toward providing tobacco cessation counseling based on the education they obtain from the medical school curriculum. In this cross-sectional study, we assessed clinical medical students' self-reported comprehensive ability to provide TCC based on the data collected from the online questionnaire. Our results showed that the self-reported comprehensive ability of clinical medical students providing TCC varied in different years of study, which is consistent with the findings of Liu et al. (19). We believe it is related to the clinical experience of the students as well as the current undergraduate tobacco medicine education in Chinese medical schools. In our study, most students realized their important role in providing TCC to patients and had a strong will to advise patients to quit tobacco smoking in their future careers. Furthermore, they generally believed that ATPs were harmful and that TCC of ATPs should be provided as well. These positive attitudes could be attributed to the professional knowledge they gained as a clinical medical student during previous stages of study and the adequate publicity on the health hazards of smoking from society. However, many students were skeptical of their ability to provide TCC and the actual effects of clinical student counseling. They also had misconceptions about the hazards of ATPs and cigarettes. In line with our findings, a recent study (19) also showed that almost all (96%) dental students had positive attitudes and perspectives on the dental professional's role in managing tobacco users in their future careers. Similar findings have been reported among medical students regarding providing tobacco cessation counseling as an effective intervention in helping patients reduce or quit smoking (20–22). Students had less confidence and perceived preparedness to provide TCC of ATPs compared to traditional cigarettes. This could be due to students' lack of knowledge about ATPs, which is consistent with the conclusion of Zhou et al. (23), suggesting that neither the concepts nor the methods of TCC were comprehensively introduced in the undergraduate medical specialized curriculum. A cross-sectional survey of tobacco habits, attitudes, and education among medical students in the United States and Italy found that Italian medical students were less likely to offer smoking cessation counseling or treatment to patients (24). This could be due to a lack of tobacco education and smoking cessation training provided to Italian medical students. Similarly, a nationwide survey of German medical students discovered that, even in their final year of undergraduate training, most students reported inadequate knowledge and were unprepared to counsel their patients to quit smoking (25). Martins et al. (26) evaluated attitudes, beliefs, and knowledge of various forms of tobacco use among medical students at a major university in Brazil and found that experimentation with water-pipe tobacco smoking and other forms of tobacco use was common among future physicians. More than half of non-smokers believed that health professionals who smoked cigarettes were less likely to advise their patients to quit smoking. The majority of the medical students were aware of the potential dangers of smoking tobacco products. Purkabiri et al. (27) found that medical students initially lacked knowledge, skills, and experience in dealing with tobacco cessation. However, a 4-h smoking intervention workshop offered by a doctoral student was highly effective in improving students' smoking cessation counseling knowledge and skills. They were also confident in providing future smoking cessation counseling to their patients. According to an Australian study (28), the number of medical schools teaching about tobacco and smoking cessation techniques has increased in recent years. Despite the fact that the majority of teaching content is based on evidence found in clinical practice guidelines, there is still much work to be done. There are barriers in developing countries, such as a lack of human and financial resources for evidence-based smoking cessation counseling and treatment (29, 30). Tobacco control leaders in medical schools should collaborate to address these issues, but they will require increased government support and capacity, as well as a significant increase in the effort to make tobacco education an integral part of medical curricula.

Of clinical relevance, statistical differences (*p* < 0.001) were found in most items assessing the self-reported comprehensive ability of clinical medical students providing TCC, and this ability varies in different years of study with an upward trend from year 1 to year 5. Undergraduates' accumulation of medical knowledge and the increasing clinical experience within the curriculum system, including the clinical practice in year 5 and clinical tutorials courses in hospitals during years 3 and 4, could account for this upward trend. Regarding the postgraduates group, it's not surprising that students in respiratory programs possessed a better ability than students in non-respiratory program. Still, they all showed a better ability than the undergraduate, because they had already completed their bachelor's degree and have received clinical skills training. A recent study (31) of the type and extent of smoking cessation advice given to patients by physicians of various medical specialties revealed that ophthalmologists, infectious disease specialists, and general surgeons were uninterested in their patients' cigarette smoking habits. Doctors in pulmonology and thoracic surgery, on the other hand, had the highest frequency of inquiring about their patients' smoking habits because they are directly interested in the respiratory system (31). The Chinese undergraduate medical education standards-clinical medicine issued by the ministry of education stipulates that in the basic standards for clinical medicine courses, medical schools must ensure that students acquire comprehensive clinical knowledge, clinical skills, and professional ability. It is recommended that undergraduate medical students begin clinical practice as soon as possible (32). Furthermore, our research found that as clinical experience increases, so does the ability to provide smoking cessation counseling. As a result, we recommend that schools carry out clinical courses and practical activities earlier so that undergraduate medical students can adapt to the clinical environment and increase their ability to provide smoking cessation counseling.

### Recommendations for comprehensive tobacco control strategies among clinical medical students in China

Historically, most smokers began smoking before the age of 18, so it is necessary to strengthen tobacco education for college students (33). Among all kinds of ways to quit smoking, smoking cessation counseling provided by doctors is more acceptable to patients. It is emphasized that medical students play an important role in providing smoking cessation counseling and tobacco-related information. Therefore, it is necessary to strengthen the tobacco education of medical students in the future. It can be seen from the training plans of various medical colleges that there is no systematic and comprehensive teaching of tobacco disease knowledge in the compulsory courses, and the students in the elective courses are not very motivated to take this course (10, 34). At the same time, it is difficult for students to apply knowledge to practice. We also believe that more professional and elective courses on tobacco education, as well as more lectures on relevant knowledge and clinical practice activities, should be established. The formulation of all courses and programs should be based on the 5A (Ask, Advise, Assess, Assist, and Arrange) smoking cessation intervention model; however, the use of 5A is limited by various factors (35). A multi-modal educational intervention may improve medical students' 5A and smoking cessation counseling ability compared with traditional educational methods (36, 37). Seminars and consulting can be easily incorporated into the medical school curriculum, enhancing students' knowledge, ability, and confidence in smoking cessation counseling. Online tobacco education training can improve learning efficiency and avoid offline gatherings during the COVID-19 pandemic. These interventions will serve as a guideline for developing a tobacco education curriculum. In addition, different medical schools can integrate tobacco education courses into other medical disciplines and teaching processes based on their training plans and current tobacco education development, based on the principles of the Global Standards for quality improvement in undergraduate medical education (38).

This study also has some limitations. The scope of this study is limited to Chongqing Medical University, and the sample size is relatively small. At the same time, we believe that graduate students have more opportunities to be exposed to the clinical environment. Hence, most of the measures we proposed are only for undergraduate students. Participants completed the survey by scanning a QR code online, so the extent of bias in online surveys cannot be overlooked, which may underestimate medical students' true attitudes and preparedness for TCC. For the research purpose of this topic, we only put forward two hypothesis factors, and more factors can be considered in future research. We believe that our study can provide directions and ideas for future tobacco education and reform.

## Conclusion

Although clinical medical students had positive attitudes toward their role in tobacco cessation promotion, few were well-prepared to manage tobacco-using patients. Therefore, future educational programs should focus on students' tobacco education and clinical course, which will enable medical students to serve as smoking cessation counselors.

## Data availability statement

The raw data supporting the conclusions of this article will be made available by the authors, without undue reservation.

## Ethics statement

The studies involving human participants were reviewed and approved by the First Affiliated Hospital of Chongqing Medical University. The Ethics Committee waived the requirement of written informed consent for participation.

## Author contributions

CY, WH, RD, HD, and MG: conceptualization, data collection, and original draft preparation. CY, WH, RD, and HD: questionnaire preparation and validation. CY, WH, and HD: data analysis, resource, and supervision. All authors have read and approved the final manuscript before submission.

## Conflict of interest

The authors declare that the research was conducted in the absence of any commercial or financial relationships that could be construed as a potential conflict of interest.

## Publisher's note

All claims expressed in this article are solely those of the authors and do not necessarily represent those of their affiliated organizations, or those of the publisher, the editors and the reviewers. Any product that may be evaluated in this article, or claim that may be made by its manufacturer, is not guaranteed or endorsed by the publisher.
